# Correlation between findings of the oral myofunctional clinical assessment, pressure and electromyographic activity of the tongue during swallowing in individuals with different orofacial myofunctional disorders

**DOI:** 10.1590/2317-1782/20232022053en

**Published:** 2023-10-09

**Authors:** Robson Rodrigues, Fernanda Chiarion Sassi, Amanda Pagliotto da Silva, Claudia Regina Furquim de Andrade

**Affiliations:** 1 Divisão de Fonoaudiologia, Hospital das Clínicas - HC, Faculdade de Medicina - FM, Universidade de São Paulo - USP - São Paulo (SP), Brasil.; 2 Departamento de Fisioterapia, Fonoaudiologia e Terapia Ocupacional, Faculdade de Medicina - FM, Universidade de São Paulo - USP - São Paulo (SP), Brasil.

**Keywords:** Speech-Language Pathology, Tongue, Deglutition, Dental Occlusion, Electromyography, Muscle Strength

## Abstract

**Purpose:**

To correlate the findings regarding the myofunctional orofacial examination, tongue pressure and surface electromyography (sEMG) of deglutition in individuals with different orofacial myofunctional disorders.

**Methods:**

44 patients (20 males and 24 females, aged between 17 and 63 years old) with different orofacial myofunctional changes were clinically assessed using the Expanded Protocol of Orofacial Myofunctional Evaluation with Scores (OMES-E). In addition, the range of mandibular movements and facial anthropometry were measured, along with the assessment of the tongue pressure (tip and dorsum) and of the electrical activity of the suprahyoid muscles during deglutition, using surface electromyography (sEMG).

**Results:**

The statistical analysis found weak correlations between tongue dorsum pressure values, suggesting that the greater the measurement of the lower third of the face, the lower the pressure of the tongue dorsum; the greater the measurement of the overlaps (vertical and horizontal), the higher the pressure of the tongue dorsum; the higher the score from the orofacial evaluation and orofacial functions assessment, the higher the pressure of the tongue dorsum; and the higher the pressure of the tongue dorsum, the higher the pressure of the tongue tip.

**Conclusion:**

The present study results indicate that the orofacial myofunctional changes found in different groups of patients are more related to the maxillomandibular discrepancies than to the pathologies investigated herein.

## INTRODUCTION

Even though all orofacial structures perform integrated actions in different orofacial functions, the tongue is identified as the most important structure for the performance of some functions, such as in the preparatory and oral phases of deglutition, being responsible for the support, manipulation, and ejection of the bolus in an anteroposterior direction for the beginning of the pharyngeal phase^(1)^.

Understanding the functioning of the tongue is fundamental to the orofacial motricity context. The tongue is formed by two distinct muscle groups: the intrinsic muscles (superior longitudinal muscle, inferior longitudinal muscle, vertical muscle and transverse muscle) and the extrinsic muscles (styloglossus, hyoglossus, genioglossus, and palatoglossus)^(1)^, being innervated by several cranial nerve pairs (V, VII, IX, and XII)^(2)^, with the first group being responsible for forming the tongue body and performing its contractions, and the second for the tongue mobility relative to the jaw^(1)^.

There are several methods to assess the human tongue. For many years, speech-language pathology assessment methods for orofacial motricity were subjective, based strictly on professional clinical experience^(3-5)^. However, with the progress of technical and scientific development, new tools have been created to complement the orofacial examination. There has been a tendency to implement methods able to quantify clinical data, allowing for the verification of the therapeutic process effectiveness, thus making the evaluation process more accurate and objective^(4)^. Among the clinical assessment methods that have been elaborated, the following techniques stand out: standardized clinical protocols with scores^(6)^, photographic documentation^(4)^, facial anthropometry performed with instruments such as the pachymeter^(7)^ and the goniometer^(4)^, as well as the use of software created to assess areas that are difficult to reach with the aforementioned instruments^(4)^.

Recently, electronic devices and more advanced technologies have also been used to complement the orofacial motricity assessments. Ultrasonography^(4,8-11)^ facilitates the analysis of different orofacial structures, such as the masseter muscle during swallowing tasks^(10)^, the tongue during speech tasks^(4)^ and the suprahyoid muscles during deglutition^(9,10,12,13)^. In turn, the surface electromyography (SSEs) assesses both the deglutition muscles and the suprahyoid muscles by placing extraoral electrodes on the skin surface to capture the muscle action potential of these regions, providing information regarding the timing of muscle activity as well as the signal amplitude^(14)^. The SSEs method is considered relevant for the evaluation of the deglutition process, producing objective data on the laryngeal elevation and anteriorization without generating risks to the patient^(15)^.

Tongue strength is an equally important form of complementary examination, objectively assessed by the maximum pressure exerted on the palate. Several devices have been studied for such a purpose, including national instruments^(16)^. These devices can be divided into four groups according to the technology used, namely: mouthpieces containing sensors; sensors fixed on the teeth, palate or palatal plates; and fluid-filled bulbs connected to pressure sensors, among other technologies. These types of equipment can all contribute to a more comprehensive orofacial motricity evaluation^(3)^.

The impairment of the pressure exerted by the tongue is highlighted as a factor associated with dysphagia and is considered to be a predictive factor for the retention of food residue in the pharyngeal region^(17)^. A previous study has provided enough scientific evidence to support the clinical use of devices to measure tongue strength. A meta-analysis with 13,773 participants showed that the maximum pressure of the tongue in individuals aged over 60 years old is significantly lower than in individuals aged less than 60 years old, therefore being related to the physiological changes of aging^(17)^. According to the literature, the brand of the equipment is a variable that interferes significantly with the maximum pressure values of the tongue, with the measurements’ discrepancies being attributed to the physical features of the air bulb. Young men and women present significant differences concerning tongue pressure, which does not occur for men and women aged over 60 years old^(17)^.

In this context, the main purpose of the present study was to correlate the findings regarding the orofacial myofunctional examination with the results of the tongue pressure assessment and the electromyographic evaluation of deglutition in groups of individuals with different orofacial changes. As a secondary objective, this study investigated whether the distinct groups of patients presented specific alterations in orofacial motricity that could detect the pathology.

## METHODS

This is a prospective cross-sectional study approved by the Research Ethics Committee of the respective institution (CAPPesq Process No. 3,799,029). The data collection only started after the signing of the Informed Consent Form (ICF) by the research participants.

### Participants

This study included patients from different health clinics of the University of São Paulo Faculty of Medicine Clinics Hospital (HC FMUSP - Hospital Clínicas of the Medical School of the University of São Paulo), referred for evaluation and possible rehabilitation at the Speech-Language Pathology Division of the same institution, between June 2018 and January 2020. The following inclusion criteria were applied: age between 18 and 63 years, no ongoing orthodontic treatment, minimum mouth opening of fifteen millimeters and presence of referential dental elements (first upper and lower molars/canine teeth, upper and lower central incisors). All data were collected in the patient’s first appointment.

The subsequent pathologies were included in this study:

- Oropharyngeal dysphagia: Score between 3 and 6 on the functional swallowing scale (ASHA NOMS)^(18)^, with ingestion of thin liquids. The presence of dysphagia was confirmed by the Speech-Language Pathologists of the HC FMUSP after the application of the Dysphagia Risk Evaluation Protocol (DREP)^(19)^.
- Facial Trauma and Orthognathic Surgery: Diagnosis of orofacial myofunctional changes, with facial trauma or post-orthognathic surgery. The referral to the speech-language pathologists was made after medical clearance.
- Obstructive Sleep Apnea/Hypopnea Syndrome (OSAHS): Diagnosis of mild (between 05 and 15 AHI/hour) to moderate (15 to 30 AHI/hour) OSAHS and no apnea surgery.
- Facial Paralysis: Unilateral peripheral facial paralysis with onset time of less than or equal to six months and House-Brackmann score^(20)^ from mild (II) to moderate-severe (IV).
- Cleft Lip and Palate: Participants with cleft lip and palate already surgically corrected. In cleft palate cases, we included post-palatoplasty patients with no incidence of fistula in the surgical region.

### Orofacial motricity assessment

We applied the OMES-E protocol^(6)^ to assess the components of the orofacial myofunctional system in terms of appearance/posture, mobility and performance during deglutition and mastication tasks. The data were collected through visual evaluation during the assessment, with the photographic and film records being analyzed using a tablet (iPad Air model A1475, Apple, USA).

In order to ensure the reliability of the clinical assessment results, all participants were evaluated by two independent speech-language pathologists with previous experience in the area. The Kappa coefficient was applied to verify the agreement between the raters, with the result indicating a high agreement rate (0.83).

The anthropometric evaluation and measurements of the mandibular amplitude^(21,22)^ were performed using a digital pachymeter (Digimess - Pró-Fono), with the following elements assessed:

- Upper face: Measurement from the trichion to the glabella.
- Mid-face: Measurement from the glabella to the subnasale.
- Lower face: Measurement from the subnasale to the gnathion.
- Midline: With teeth in occlusion, the distance between the upper and the lower central incisor lines.
- Horizontal overlap: With teeth in occlusion, the distance between the side of the upper central incisor and the side of the lower central incisor.
- Vertical overlap: With teeth in occlusion, the distance between the edge of the lower central incisor and the edge of the upper central incisor.
- Maximum mouth opening: Distance between the incisal surfaces of the central (upper and lower) incisors, including the vertical overlap measurement.
- Jaw lateralization: Horizontal distance between the upper and lower incisor lines after lateral jaw sliding (to the right and then to the left), performing the appropriate adjustments in cases of midline deviation of the central incisors.
- Jaw protrusion: Horizontal distance between the buccal surface of the central incisors (upper and lower) after anterior sliding, including the horizontal overlap measurements.

### Assessment of the suprahyoid musculature - Surface Electromyography (SSEs)

The SSEs tests were conducted by the same speech-language pathologist in the same environmental conditions. The electromyographic evaluation of the participants’ suprahyoid muscles was performed based on a specific methodology^(23)^. A 4-channel equipment (Miotool 400) was used for the assessments, with the following calibration: at 500 microvolts (μV) with a bandpass filter (20-500 Hz) coupled with a notch filter (60 Hz) and 100x gain, with low noise level (< 5μV RMS). SSEs were captured and processed using the Miograph 2.0 app from the manufacturer Miotec® Equipamentos Biomédicos, which allows for an online acquisition, storage and processing of signals and runs under the Windows XP operating system or newer. The electrical activity signals of the muscle movements were captured with disposable double bipolar surface electrodes Ag/AgCl, model SDS500, fixed with transpore tape (3M).

The electrodes were placed directly on the skin, previously cleaned with 70% alcohol following hair removal, using the technique of placing the midpoint of the muscle belly in the longitudinal direction of the fasciculus in the mesiodistal position of the muscle, where there is a greater signal amplitude for this type of electrode. As suggested by Soderberg and Cook^(24)^, the assessment was performed after placing the electrodes, repositioning if necessary. The captured signals were analyzed in root mean square (RMS) and expressed in microvolts (μV). The reference cable (ground wire) was connected to the electrode and fixed over the right wrist.

The participants remained comfortably seated on a chair during the data collection, with their backs supported, feet flat on the floor, hands resting on the lower limbs, heads properly positioned (Frankfurt horizontal plane, parallel to the floor), eyes open and looking at a predetermined fixed point. All the individuals received instructions for the test.

The electromyographic evaluation was carried out in three steps with three-minute breaks between assessments:

- Rest: Each participant was instructed to remain as relaxed as possible for one minute. Afterwards, three 30-second recordings of the suprahyoid muscle activity were conducted.
- Assessment of room temperature water deglutition: All participants were given the following instruction - “Drink all the water in just one sip.” All data were collected in 15-second recordings. The subsequent tests were performed three times:
  - Voluntary swallowing of saliva: Participants were given the following instruction: “Swallow the saliva that is currently in your mouth.”
  - Sampling of 10 ml of water with the syringe.
  - Sampling of 16.5 ml of water with the syringe.
  - Sampling of 20 ml of water with the syringe.
- Assessment of deglutition with a larger volume (50ml) and self-managed sampling with a cup: All participants received the following instruction: “Drink all the water as you usually do, while keeping this same head posture”. All data were collected in 15-second recordings.

The analysis of the electromyographic results considered the signal amplitude. In the resting scenario, the obtained values represented the mean (RMS) electromyographic activity observed for 30 seconds. The time of muscle activity during the deglutition tasks was assessed by selecting the duration of the muscle activation (on, peak and off settings).

The well-known variability in the electromyographic signal^(25)^ requires the use of data normalization techniques for group comparison, which is a way to transform absolute amplitude values into relative values referring to an amplitude value characterized as 100%. The technique used in this study was the normalization of activities concerning the resting tasks.

The reliability analysis was conducted to determine the agreement limits between raters and ensure a greater consistency of the measurements. In order to do so, 50 electromyographic samples referring to the deglutition tasks were selected from a total of 615 samples available (three participants presented electromyographic signals with artifacts, thus disabling the data collection). These samples were independently analyzed by two experienced speech-language pathologists blinded to the study. The correlation coefficient was high for all comparisons (confidence interval of 95% [CI] = 0,8345-0,9158), indicating a high agreement among raters.

### Tongue Pressure Measurement (PLL Pró-Fono):

The Biofeedback Pró-Fono - Lips and Tongue Pressure Measurement (PLL Pró-Fono) is an instrument validated by the Brazilian Health Regulatory Agency (Anvisa) for clinical use in Brazil. The PLL Pró-Fono consists of a pressure sensor connected to an electronic board, packed in a plastic enclosure. Within this enclosure is attached a flexible plastic air tube, which receives a disposable bulb with a male connector plug on its end for tongue pressure collection, according to the technical manual instructions. The information concerning pressure variation exerted on the bulb is sent by the device to the equipment’s software installed in the computer, providing real-time visual feedback through graphs. The PLL Pró-Fono software must be previously installed on the computer, which requires the Windows XP operating system or newer.

The pressure value of the dorsum and tip of the tongue was assessed pursuant to the following instructions:

- Tongue dorsum pressure: The patient was asked to hold the air bulb tube with one hand, open their mouth and place it on the back of the tongue, so that the end of the air bulb attached to its tube was positioned on the tip of the participant’s tongue, with the pressure between the dorsum of the tongue and the palate was applied on the sides of the air bulb. The patient could slightly occlude their lips, provided that there was no contact or pressure between the teeth, being instructed and encouraged to press the entire length of the tongue on the air bulb against the palatal region for 5 seconds, with a 30-second break. Three collections were performed and, in each break, the patient was allowed to swallow the saliva and dry the oral cavity with gauze to prevent the bulb from slipping during the exam.
- Tongue tip pressure: The participant was asked to hold the air bulb tube with one hand, open their mouth and place the air bulb on the tip of the tongue so that the tip of the air bulb attached to its tube was positioned on the dental alveoli of the patient’s central incisor teeth, with the pressure between the tip of the tongue and the palate applied on the hemifaces of the air bulb. The participant could slightly occlude their lips, provided that there was no contact or pressure between the teeth, being instructed and encouraged to press the entire tip of the tongue on the air bulb against the palatal papilla for 5 seconds, with a 30-second break. Three collections were performed and, in each break, the patient was allowed to swallow the saliva and dry the oral cavity with gauze to prevent the bulb from slipping during the exam.

## DATA ANALYSIS

The collected data underwent a statistical analysis on the SPSS software version 28.0, being subjected to two forms of analysis. Initially, the data received a descriptive analysis to characterize the sample: the quantitative variables were described by mean, standard deviation, median, minimum and maximum values, while the qualitative variables were described by total count and percentage. The Spearman’s Rank correlation coefficient was applied for the inferential analysis, in order to detect the presence of a correlation between the tongue pressure measures (dorsum and tip) and the other variables studied. This non-parametric test is a viable and adequate alternative for this type of analysis, due to requiring neither normality nor equality of variances and being applied to small samples. The data interpretation of the correlation analysis used the following parameters: strong negative relationship (r < -0.750); moderate negative relationship (-0.750 < r < -0.500); weak negative relationship (-0.500 < r < -0.250); low or no relationship (-0.250 < r < 0.250); weak positive relationship (0.250 < r < 0.500); moderate positive relationship (0.500 < r < 0.750), and strong positive relationship (r > 0.750).

## RESULTS

Table 1 describes the patients’ sample characterization regarding the population and the clinical variables. The participants were aged on average 34.7 years and the group with facial paralysis diagnosis had the highest number of patients, followed by cleft lip and palate. In addition, most participants presented an Angle Class I bite classification.

**Table 1:** Sample characterization according to demographic and clinical data (n=44)

	**Results**
**Age, in years**	
valid n (available data)	44
mean (±SD)	34.7 (±13.4)
median (min; max)	35.0 (17; 63)
**Sex, n (%)**	
Male	20 (45.5%)
Female	24 (54.5%)
**Pathology, n (%)**	
Facial Paralysis	13 (29.5%)
Cleft Lip and Palate	11 (25.0%)
Facial Trauma	6 (13.6%)
OSAHS	3 (6.8%)
Dysphagia	9 (20.5%)
Orthognathic surgery	2 (4.5%)
**Angle Occlusion, n (%)**	
Class I	35 (79.5%)
Class II	2 (4.5%)
Class III	7 (15.7%)

**Caption:** n: number of participants; SD: standard deviation; min.: minimum; max.: maximum; OSAHS: Obstructive Sleep Apnea/Hypopnea Syndrome

Table 2 describes the data of the facial anthropometric measurements and the dynamic jaw amplitude assessments, as well as the OMES-E protocol scores. For comparison purposes, the normality measurements described in the literature^(3,20,21)^ were included in this table. The present study found a discrepancy between the participants’ facial thirds, with the lower face being more elongated. As to the jaw amplitude assessments, there was a greater impairment of the jaw lateralization and the jaw protrusion measurements, in addition to the vertical overlapping.

**Table 2:** Sample characterization according to facial measurements, dynamics, and OMES-E protocol (n=44)

		**Measure, in mm**		**Normality, in mm**
		**mean (±SD)**	**median (min; max)**	**mean (±SD)**
**Facial and jaw measurements**	**Upper face**	58.5 (±7.6)	58.0 (41.6; 72.6)	55.00 - 65.00
	**Mid-face**	58.2(±4.6)	58.3 (48.6; 66.9)	55.00 - 65.00
	**Lower face**	65.6 (±6.7)	66.1 (53.1; 86.4)	55.00 - 65.00
	**Full opening**	45.0 (±9.9)	46.7 (9.2; 58.5)	40.00 - 60.00
	**Jaw lateralization - right side**	6.0 (±3.1)	6.2 (0.0; 13.9)	7.00 - 11.00
	**Jaw lateralization - left side**	6.1 (±3.0)	6.2 (0.1; 13.6)	7.00 - 11.00
	**Jaw protrusion**	6.5 (±2.6)	6.5 (0.0; 11.1)	7.00 - 11.00
	**Horizontal overlap**	2.8 (±4.0)	3.5 (-13.7; 9.1)	2.00 - 3.00
	**Vertical overlap**	2.7 (±2.5)	2.8 (-7.8; 7.3)	1.00 - 2.00
		**Patient’s score**		**Maximum Score Possible**
		**mean (±SD)**	**median (min; max)**	
**OMES-E**	**Appearance and postural condition/position - total**	53.1 (±4.1)	53.0 (45; 61)	64
	**Mobility - total**	91.2 (±17.7)	91.0 (52; 160)	114
	**Functions - total**	42.3 (±5.6)	42.0 (29; 55)	52
	**AMIOFE - final score**	184.4 (±20.9)	184.0 (134; 258)	230

**Caption:** n: number of participants; mm: millimeters; SD: standard deviation; min.: minimum; max.: maximum; OMES-E: Expanded Protocol of Orofacial Myofunctional Evaluation with Scores

Regarding the OMES-E, Table 2 includes the maximum score possible, according to the test. Based on this analysis, considering the mean score obtained by the participants, it is possible to observe that the sample group reached 82.9% of the expected score for appearance and posture condition, 80% for mobility, 81.3% for functions, amounting to a total of 80.2%.

Table 3 describes the results of the electromyographic evaluation of the suprahyoid muscles at resting and at deglutition of both saliva and controlled volumes of water (10; 16.5; 20, and 50 ml). It is worth mentioning that the number of peaks found for the swallowing of saliva and volumes of water corresponds to the number of deglutition events performed by the participants to swallow each volume. As expected, the muscle activation/recruitment during deglutition was clearer for the swallowing of 50ml of water, requiring a longer time to finish the task and a higher number of deglutition events to completely finish the volume supplied.

**Table 3:** Sample characterization according to the results of the electromyographic assessment (n=41)

**Variable**			**SSE results**		
			Valid n	Mean (SD)	Median (min;max)
Rest: electromyographic activity, in μV (RMS)		Right side	41	5.2 (±6.2)	3.6 (0.0; 33.9)
		Left side		3.8 (±2.9)	3.4 (0.0; 14.5)
Time, in seconds		Deglutition of saliva	41	1.0 (±0.5)	1.1 (0.0; 2.2)
		Deglutition of 10ml of water		1.1 (±0.6)	1.0 (0.0; 3.3)
		Deglutition of 16.5ml of water		1.0 (±0.5)	1.0 (0.0; 2.3)
		Deglutition of 20ml of water		1.1 (±0.5)	1.1 (0.0; 2.9)
		Deglutition of 50ml of water		8.0 (±4.2)	7.8 (0.0; 16.7)
Number of peaks		Deglutition of saliva	41	1.0 (±0.5)	1.0 (0.0; 2.0)
		Deglutition of 10ml of water		1.1 (±0.5)	1.0 (0.0; 2.0)
		Deglutition of 16.5ml of water		1.0 (±0.4)	1.0 (0.0; 1.7)
		Deglutition of 20ml of water		1.1 (±0.4)	1.0 (0.0; 2.0)
		Deglutition of 50ml of water		4.5 (±2.3)	4.3 (0.0; 10.7)
Normalized measure: electromyographic activity, in μV (RMS)	Right side	Deglutition of saliva	41	7.5 (±4.0)	6.9 (1.2; 18.3)
		Deglutition of 10ml of water		8.2 (±5.0)	7.1 (0.0; 20.8)
		Deglutition of 16.5ml of water		8.5 (±5.1)	8.1 (0.0; 21.2)
		Deglutition of 20ml of water		9.2 (±4.9)	10.0 (1.2; 22.7)
		Deglutition of 50ml of water		6.3 (±3.6)	6.2 (0.0; 15.3)
	Left side	Deglutition of saliva		81 (±4.0)	8.2 (1.8; 18.3)
		Deglutition of 10ml of water		8.9 (±4.7)	8.6 (0.0; 21.3)
		Deglutition of 16.5ml of water		9.1 (±4.7)	8.8 (0.0; 20.1)
		Deglutition of 20ml of water		9.6 (±4.3)	9.8 (1.7; 22.6)
		Deglutition of 50ml of water		6.7 (±3.2)	6.9 (0.0; 14.0)

**Caption:** n: number of participants; SD: standard deviation; min.: minimum; max.: maximum; SSE: surface electromyography; RMS: root mean square; μV: microvolts

Table 4 describes the participants’ sample characterization concerning the tongue pressure assessment results. Tongue pressure proved to be similar between dorsum and tip values.

**Table 4:** Sample characterization according to tongue pressure assessment (n=44)

	**Measurement, in kPa**		
	valid n (available data)	mean (±SD)	median (min; max)
**Tongue dorsum**	43	33.6 (±14.3)	32.8 (0.0; 68.6)
**Tongue tip**		33.6 (±12.0)	33.5 (10.6; 62.7)

**Caption:** n: number of participants; SD: standard deviation; min.: minimum; max.: maximum; kPa: Kilopascal

Table 5 presents the results of the correlation analysis between the tongue pressure assessment data and the other studied variables. Significant correlations were found between the tongue dorsum pressure and the lower face measurements; between the tongue dorsum pressure and the vertical and horizontal overlap measurements; between the tongue dorsum pressure and the appearance and posture condition scores according to the OMES-E protocol^(3)^; between the tongue dorsum pressure and the functions according to the OMES-E protocol score^(3)^; and between the tongue dorsum and tip pressures. It is noteworthy that all correlations found herein were weak and that none of the individuals in the sample group was able to proceed with the collection due to hypersensitivity with persistent nauseous reflex during the examination attempts.

**Table 5:** Correlations between the results of the tongue pressure assessment and the other variables of the sample characterization

		**Correlations with tongue pressure assessment**			
		**Tongue dorsum**		**Tongue tip**	
		**r**	**p-value**	**r**	**p-value**
Age		-0.125	0.426	-0.241	0.120
Sex		0.103	0.513	-0.054	0.731
Pathology		-0.146	0.352	0.068	0.665
Measurement	Upper face	0.071	0.650	0.060	0.702
	Mid-face	0.069	0.662	0.094	0.550
	Lower face	***-0.306***	0.046*	-0.018	0.911
	Occlusion angle	-0.159	0.310	0.265	0.086
	Full opening	0.121	0.470	-0.092	0.581
	Midline deviation	-0.053	0.747	0.067	0.687
	Lateralization - right	-0.083	0.620	-0.301	0.067
	Lateralization - left	0.110	0.511	0.076	0.650
	Jaw protrusion	0.240	0.152	-0.214	0.203
	Jaw retraction	-0.198	0.241	0.033	0.847
	Horizontal overlap	***0.329***	0.047*	-0.108	0.525
	Vertical overlap	***0.418***	0.010*	-0.038	0.824
AMIOFE score	Appearance and posture condition	***0.471***	0.001*	0.036	0.817
	Mobility	0.014	0.930	0.031	0.842
	Functions	***0.365***	0.016*	0.113	0.471
	Total score	-0.001	0.994	-0.145	0.355
SSE evaluation	Resting activity - right	0.139	0.375	-0.057	0.715
	Resting activity - left	0.176	0.259	-0.016	0.919
	Deglutition time - saliva	0.132	0.398	-0.013	0.935
	Deglutition time - 10ml	-0.157	0.315	-0.034	0.830
	Deglutition time - 16.5ml	-0.156	0.317	-0.024	0.878
	Deglutition time - 20ml	0.086	0.582	0.035	0.822
	Deglutition time - 50ml	-0.279	0.070	-0.203	0.193
	Number of peaks - saliva	0.206	0.184	0.147	0.347
	Number of peaks - 10ml	-0.212	0.172	0.022	0.890
	Number of peaks - 16.5ml	-0.125	0.425	0.052	0.738
	Number of peaks - 20ml	-0.007	0.967	-0.053	0.738
	Number of peaks - 50ml	-0.265	0.085	-0.056	0.722
	Maximum peak - saliva - right	***0.355***	0.019*	0.192	0.217
	Maximum peak - 10ml - right	-0.103	0.509	0.063	0.689
	Maximum peak - 16.5ml - right	-0.091	0.563	0.069	0.659
	Maximum peak - 20ml - right	-0.017	0.916	0.065	0.677
	Maximum peak - 50ml - right	0.021	0.892	-0.047	0.767
	Maximum peak - saliva - left	***0.329***	***0.031*** *	0.167	0.285
	Maximum peak - 10ml - left	-0.118	0.451	0.119	0.445
	Maximum peak - 16.5ml - left	-0.118	0.452	0.105	0.503
	Maximum peak - 20ml - left	-0.050	0.748	0.110	0.482
	Maximum peak - 50ml - left	-0.082	0.599	0.078	0.618
	Normalized - saliva - right	0.185	0.247	0.169	0.292
	Normalized - 10ml - right	0.059	0.715	0.116	0.469
	Normalized - 16.5ml - right	0.032	0.844	0.103	0.522
	Normalized - 20ml - right	0.125	0.435	0.088	0.585
	Normalized - 50ml - right	0.129	0.421	0.107	0.504
	Normalized - saliva - left	0.034	0.835	-0.010	0.953
	Normalized - 10ml - left	-0.045	0.780	-0.037	0.817
	Normalized - 16.5ml - left	-0.069	0.668	-0.057	0.723
	Normalized - 20ml - left	0.007	0.965	-0.085	0.598
	Normalized - 50ml - left	0.000	1.000	-0.018	0.910
Measurement of the tongue dorsum pressure (TLP)		-	-	***0.490***	<0.001*

* statistically significant difference, according to the Spearman’s Rank correlation coefficient

**Caption:** r: correlation coefficient; SSE: surface electromyography; TLP: tongue and lips pressure

## DISCUSSION

Overall, the results analysis indicated the following significant correlation between the instruments used to characterize the participants’ sample group: the greater the horizontal overlapping, the greater the tongue pressure, and the higher the score in the myofunctional assessment for rest and orofacial functions; and the longer the lower face measurement, the lower the tongue dorsum pressure.

Although almost the entire sample of patients in this study presented a Class I occlusion, some participants showed Class II and III occlusions^(26)^, which interfered with the results of the orofacial motricity evaluation, as well as with the anthropometric assessments and jaw amplitude measurements. Several studies^(27-29)^ have been carried out correlating the stomatognathic system’s form and function.

For Class III malocclusions, the literature indicates frequent masticatory alterations in this population, with predominantly vertical movements and use of the tongue dorsum to knead the food; flaccid and half-open lips when resting; flaccid and enlarged tongue on the buccal floor^(27,30)^. As observed in the present study, these were the most impaired areas according to the orofacial motricity evaluation of the participants in this research. The higher occurrence of Class III malocclusion in the group with cleft lip and palate is associated to the jaw growth and protrusion throughout the development phase, as well as to mandibular hypoplasia, which results from congenital bone alteration and multiple surgical interventions in the region^(31)^.

Class II and III malocclusions showed alterations in the measurements of both the horizontal and the vertical overlaps, with a much lower horizontal overlapping for the Class III malocclusion. In addition, in more severe cases, there was an inversion of the typical maxillomandibular relationship. According to the findings herein, these patients tend to present a lower tongue strength with a more reduced horizontal overlap, which corroborates a previous study^(3)^ that also analyzed tongue strength. According to the authors, individuals with Class III malocclusion presented a lower tongue strength and a longer pressure time during the deglutition of homogeneous pasty food when compared with the control subjects with Class I occlusion and no previous orofacial complaints or interventions^(32)^. It should be highlighted that there is no uniformity regarding the data related to tongue pressure alterations and malocclusions in the literature. Silva et al.^(33)^ found no significant relationship between tongue strength and the different types of dental occlusion. Based on the present study findings, a possible explanation for the lower tongue pressure observed in individuals with Class III malocclusion is the jaw protrusion peculiar to this population, resulting in a larger intraoral space and demanding a larger amplitude of tongue movements to perform counter-resistance tasks in the palate area (pressure)^(34)^. Even though a previous study suggested that this type of occlusion (Angle I, II, and III) does not interfere with the tongue strength^(33)^, it is known that in Class III malocclusions both the tongue and the hyoid bone are anteriorized^(28)^. By performing deglutition movements, the tongue of individuals with Class III malocclusion follows the same pattern of movements observed in Class I, but with lower strength and requiring a longer time to perform the same activity, making the function less effective^(28)^.

The facial disproportion observed in the participants is also remarkable, since the lower face was usually more elongated. Once again, such a result can be explained by the interference of the data obtained by the group of patients with Class III malocclusion. According to the literature, this population tends to present longer face features (dolichofacial), with flaccidity of the orofacial muscles leading to alterations of the orofacial functions, mainly masticating and breathing^(26)^.

The myofunctional orofacial examination system using the OMES-E protocol^(32)^ presented maximum scores close to 80% in all areas of analysis, with two presenting a positive correlation with tongue dorsum strength: the posture condition and the orofacial functions (breathing, masticating and deglutition are covered by the protocol). The presence of tongue strength in the myofunctional assessment corroborates the hypothesis that the orofacial structures and their functions are correlated^(27,35)^. This is also true for different tongue regions. The present study results indicated no difference between the tongue tip and dorsum pressures. Another study^(24)^ used a dynamometer to measure the pressure of the tongue tip and dorsum, finding a different result: the tip of the tongue presented a lower force than the dorsum. As stated by the authors, such divergence might be explained by the presence of several types of muscle fibers in each of the tongue regions - type II fibers on the tip, ensuring a faster contraction, and type I fibers on the dorsum, providing a slower and more resistant contraction. Conversely, another study^(28)^ used palate sensors to check tongue pressure and described that the tip of the tongue is the first portion of the organ to press on the hard palate and one of the last to relax after deglutition, keeping the pressure fixed on the palatine papilla. It is worth mentioning that the methodological variability of the foregoing studies and the use of instruments to measure tongue pressure hampers the comparison and generalization of results.

The swallowing function, assessed by a SSEs of suprahyoid muscles, presented no variation of time or number of peaks in the controlled deglutition (saliva, 10 ml, 16.5 ml, and 20 ml). Different values were observed only in swallowing in self-managed sips, due to the possibility of fractioning the content and ingesting it in smaller volumes. A bilaterally increasing curve was found in the maximum electromyographic peak values as well as in the normalized measurements, indicating that the recruitment of muscle fibers increases with the volume to be ingested. Such a finding is contrary to recent researches that used magnetic articulography^(35,36)^. In one of these studies^(35)^, palate sensors were associated with the measurement of tongue pressure during swallowing of different volumes of water (3 and 10 ml). A change was detected in the movement of the tongue dorsum with lower bolus volumes before deglutition. The authors indicate that this higher activation of the tongue muscles is related to the rotation of the tongue dorsum to place the small bolus on the center of the tongue for swallowing. The authors also mentioned that there is a tendency to perform a lower recruitment of the tongue muscles for the task as the volume increases, since larger volumes adapt to the center of the tongue more easily. This study found no significant increase in the pressure exerted by the tongue on the palate^(36)^.

Only the swallowing of saliva (at a lower volume) presented a statistically significant correlation to tongue strength. A study that evaluated the dentofacial morphology and tongue function during deglutition in young individuals^(27)^ concluded that the deglutition of saliva is the function with the greatest effect on dentofacial morphology, which might explain the present study findings on the swallowing of saliva.

It is worth highlighting that all methods of standardized evaluation present a considerable advantage for the therapeutic process: the possibility of establishing intra and intersubject comparisons. Furthermore, it is also worth noting that the orofacial motricity evaluation is the most practical as well as most accessible method to clinical speech-language pathologists, and its findings must be correlated to other forms of assessment.

Finally, this study had some limitations. As aforementioned, the small number of participants, the heterogeneity of pathologies and the absence of a control group limited the generalization of results. The equipment used to verify tongue pressure was another limitation. During data collection, the patients presented difficulties to maintain the air bulb (used to measure the pressure) stable within the oral cavity. In addition, the bulb showed to be large in relation to the intraoral space, leading to the experience of nausea by many patients, which interfered with the data collection. The PLL provides the mean of tongue pressure in five seconds and not the maximum peak of pressure, as does other equipment available in the market for the same end. This fact made it impossible to compare this study findings to the results found in the literature by similar researchers. Further studies should include a larger number of participants divided by the type of malocclusion to confirm the results herein.

## CONCLUSION

The present study found the following significant correlation between the instruments used for the characterization of the participants sample group: the greater the horizontal overlap, the greater the tongue pressure and the higher the score in the myofunctional assessment for resting and orofacial functions. Additionally, the larger the measurement of the lower face, the lower the tongue dorsum pressure. The results also suggest that the orofacial myofunctional changes found among the distinct groups of patients are more related to maxillomandibular discrepancies than to the researched pathologies per se.
