# Supplementary material for: Correlation between findings of the oral myofunctional clinical assessment, pressure and electromyographic activity of the tongue during swallowing in individuals with different orofacial myofunctional disorders
Source: Codas. 2023 Oct 9;35(6):e20220053. doi: 10.1590/2317-1782/20232022053en (PMC10702706; doi:10.1590/2317-1782/20232022053en)
Supplement: Translation

# Translation: Correlação entre achados da avaliação clínica miofuncional orofacial, a pressão e a atividade eletromiográfica da língua na deglutição em indivíduos com diferentes alterações da motricidade orofacial

**DOI:** 10.1590/2317-1782/20232022053pt

**Authors:** Robson Rodrigues, Fernanda Chiarion Sassi, Amanda Pagliotto da Silva, Claudia Regina Furquim de Andrade

**Affiliations:** 1 Divisão de Fonoaudiologia, Hospital das Clínicas - HC, Faculdade de Medicina - FM, Universidade de São Paulo - USP - São Paulo (SP), Brasil.; 2 Departamento de Fisioterapia, Fonoaudiologia e Terapia Ocupacional, Faculdade de Medicina - FM, Universidade de São Paulo - USP - São Paulo (SP), Brasil.

**Keywords:** Fonoaudiologia, Língua, Deglutição, Oclusão Dentária, Eletromiografia, Força Muscular

## Abstract

**Objetivo:**

correlacionar os achados da avaliação clínica miofuncional orofacial, pressão de língua e da eletromiografia de superfície (EMGs) da deglutição de grupos de pacientes com diferentes alterações da motricidade orofacial.

**Método:**

44 pacientes (20 homens e 24 mulheres com idades entre 17 e 63 anos), com diferentes alterações miofuncionais orofaciais foram avaliados por meio da Avaliação Miofuncional Orofacial com Escores Expandido (AMIOFE-E), avaliação da amplitude mandibular e antropometria facial, mensuração da pressão de língua (ponta e dorso) e exame de Eletromiografia de Superfície (EMGs) em região supra hioidea na tarefa de deglutição de saliva e diferentes volumes de água.

**Resultados:**

a análise estatística encontrou algumas correlações fracas que envolvem a pressão do dorso de língua e sugerem que quanto maior for a medida do terço inferior, menor será a pressão do dorso da língua; quanto maior for a medida dos trespasses (vertical e horizontal) maior será a pressão do dorso da língua; quanto maior for a pontuação da avaliação de postura e funções orofaciais, maior será a pressão do dorso de língua e quanto maior for a pressão do dorso de língua, maior será a pressão da ponta da língua.

**Conclusão:**

os resultados sugerem que as alterações miofuncionais orofaciais encontradas nos diferentes grupos de pacientes estão mais relacionadas às discrepâncias maxilomandibulares do que às patologias pesquisadas no presente estudo.

## INTRODUÇÃO

Embora todos as estruturas orofaciais desempenhem ações integradas nas diferentes funções orofaciais, a língua é apontada como a estrutura mais importante de algumas funções, como nas fases preparatória e oral da deglutição, sendo responsável pela sustentação, manipulação e ejeção do bolo em sentido anteroposterior para início da fase faríngea^(1)^.

Compreender o funcionamento da língua é essencial na motricidade orofacial. Ela é formada por dois grupos musculares distintos: os intrínsecos (músculo longitudinal superior, músculo longitudinal inferior, músculo vertical e músculo transverso) e os extrínsecos (estiloglosso, o hioglosso, o genioglosso e o palatoglosso)^(1)^, inervados por vários pares cranianos (V, VII, IX e XII)^(2)^ sendo o primeiro grupo responsável por formar o corpo da língua e realizar as suas contrações e o segundo pela mobilidade da língua em relação à mandíbula^(1)^.

Existem diversas maneiras de se avaliar a língua humana. Por muitos anos, os métodos de avaliação fonoaudiológica da motricidade orofacial foram subjetivos, ou seja, baseados estritamente na experiência clínica do profissional^(3-5)^.Entretanto, com o desenvolvimento técnico e científico, novas ferramentas têm sido criadas para complementação da avaliação orofacial. Existe uma tendência à implementação de métodos que quantifiquem dados clínicos, permitindo a verificação da efetividade dos processos terapêuticos, o que torna o processo avaliativo preciso e objetivo^(4)^. Dentre os métodos de avaliação clínica que têm sido elaborados, destacam-se os protocolos clínicos padronizados com escore^(6)^,documentação fotográfica^(4)^, antropometria facial realizada por instrumentos como o paquímetro^(7)^ e o goniômetro^(4)^, além de *softwares*, que foram criados para avaliação de áreas de difícil acesso com os instrumentos citados^(4)^.

Mais recentemente, dispositivos eletrônicos e com tecnologia mais avançada também podem ser utilizados para complementar a avaliação da motricidade orofacial. A ultrassonografia^(4,8-11)^,permite analisar diferentes estruturas orofaciais, como o músculo masseter durante a tarefa de mastigação^(10)^,a língua em tarefas de fala^(4)^ e músculos supra-hioideos durante a função de deglutição^(9,10,12,13)^.Outro dispositivo utilizado é a eletromiografia de superfície (EMGs),que pode avaliar tanto os músculos mastigatórios quanto músculos supra-hioideos, por meio da colocação extraoral dos eletrodos na superfície da pele para a captação dos potenciais de ação dessas regiões musculares, fornecendo assim, informações quanto ao tempo da atividade muscular, bem como a amplitude do sinal^(14)^. A EMGs é um método considerado relevante para a avaliação da deglutição, por fornecer dados objetivos sobrea elevação e anteriorização laríngea e sem gerar riscos ao paciente^(15)^.

Outra forma relevante de avaliação complementar diz respeito à força da língua, avaliada objetivamente pela pressão máxima exercida sobre o palato. Diversos equipamentos para tal objetivo já foram estudados, inclusive os de origem nacional^(16)^. Esses equipamentos podem ser divididos em quatro grupos de acordo com a tecnologia utilizada: bocal contendo sensores; sensores fixados nos dentes, palato ou em placas palatais; bulbos preenchidos com fluidos e conectados a sensores de pressão; e outras tecnologias. Todos estes tipos de equipamentos podem contribuir para uma avaliação da motricidade orofacial mais completa^(3)^.

O comprometimento da pressão exercida pela língua é apontado como um fator associado à disfagia e é considerado um fator preditivo de resíduo alimentar em região faríngea^(17)^. Estudo anterior mostrou que existe evidência científica suficiente para sustentar a utilização clínica de aparelhos para a mensuração da força de língua; considerando uma meta-análise com 13773 participantes no total, a pressão máxima de língua em sujeitos com mais de 60 anos se apresenta significativamente menor do que em sujeitos com menos de 60 anos e está relacionado às mudanças fisiológicas do envelhecimento^(17)^. Segundo a literatura, a marca do equipamento é uma variável que interfere significativamente nos valores de pressão máxima de língua, sendo que as características físicas do bulbo de ar são apontadas como justificativa para a diferença entre as medidas. Homens jovens e mulheres jovens apresentam diferença significativa quanto a pressão de língua, mas o mesmo não acontece com homens e mulheres com mais de 60 anos^(17)^.

Diante do exposto, o objetivo geral do presente estudo foi correlacionar os achados da avaliação clínica miofuncional orofacial com resultados da avaliação de pressão de língua e da avaliação eletromiográfica da deglutição em grupos de indivíduos com diferentes alterações orofaciais. Como objetivo secundário, o estudo investigou se os diferentes grupos de pacientes apresentam alterações específicas da motricidade orofacial, capazes de identificar a patologia.

## MÉTODO

Estudo transversal prospectivo. Esta pesquisa foi aprovada pelo Comitê de Ética em Pesquisa da Instituição (CAPPesq Processo no. 3.799.029). Os procedimentos para coleta de dados tiveram início após assinatura do Termo de Consentimento Livre e Esclarecido (TCLE) pelos participantes da pesquisa.

### Participantes

Participaram do presente estudo indivíduos encaminhados pelas diferentes clínicas médicas do Hospital Clínicas da Faculdade de Medicina da Universidade de São Paulo (HC FMUSP) para avaliação e possível reabilitação fonoaudiológica na Divisão de Fonoaudiologia da mesma instituição, no período entre junho de 2018 e janeiro de 2020. Os critérios de inclusão foram: idades entre 18 e 63 anos; ausência de tratamento ortodôntico em andamento; abertura oral mínima de 15 milímetros e presença de elementos dentários referenciais (1º molar/canino superior e inferior, incisivos centrais superior e inferior). A coleta de dados foi realizada na primeira consulta do paciente.

Dessa forma, as patologias incluídas neste estudo foram:

- Disfagia Orofaríngea - escore entre 3 e 6 na escala funcional da deglutição (ASHA NOMS)^(18)^, com ingesta de líquidos finos. A presença de disfagia foi confirmada pela equipe da Divisão de Fonoaudiologia do HC FMUSP após aplicação do Protocolo Fonoaudiológico de Avaliação do Risco da Disfagia (PARD)^(19)^.
- Trauma de Face e Cirurgia Ortognática - diagnóstico de alterações miofuncionais orofaciais, com trauma de face ou em pós-operatório de cirurgia ortognática. O encaminhamento para a equipe de Fonoaudiologia foi realizado após liberação médica.
- Síndrome Apnéia e Hipopnéia Obstrutiva do Sono (SAHOS) - diagnóstico de SAHOS de leve (entre 05 a 15 IAH/hora) a moderada (15 a 30 IAH/hora) e sem realização de cirurgias para redução da apneia.
- Paralisia Facial - paralisia facial periférica unilateral e com tempo de instalação de menor ou igual a 6 meses e escala de *House Brackmann* ^(20)^ de leve (II) à moderada-grave (IV).
- Grupo Fissura Labiopalatina - participantes com fissura labiopalatina já corrigida cirurgicamente. Nos casos de fissura palatina foram incluídos participantes pós-palatoplastia e sem incidência de fístula em região cirúrgica.

### Avaliação clínica da motricidade orofacial

Foi realizada a aplicação do protocolo AMIOFE-E^(6)^ que avalia os componentes do sistema miofuncional orofacial em termos de aspecto/postura, mobilidade e desempenho durante as funções de respiração, deglutição e de mastigação. A coleta de dados foi realizada por meio de inspeção visual durante a avaliação e pela análise dos registros das fotos e filmagens, em *tablet* (IPad Air modelo A1475, Apple USA).

Com o objetivo de garantir a fidedignidade dos resultados da avaliação clínica, todos os participantes foram avaliados por dois fonoaudiólogos, examinadores independentes, com experiência na área. O Coeficiente de Kappa foi utilizado para verificar a concordância entre os examinadores, sendo que o resultado indicou alto nível de concordância (0,83).

A avaliação das medidas antropométricas e da amplitude mandibular^(21,22)^ foram realizadas com a medição por paquímetro digital (Digimess - Pró-Fono) e os aspectos mensurados foram:

- Terço superior da face: medida do trichion à glabela.
- Terço médio da face: medida da glabela ao subnasal.
- Terço inferior: medida do subnasal ao gnátio.
- Linha média: com os dentes em oclusão, distância entre as linhas dos incisivos centrais superiores e inferiores.
- Trespasse horizontal: distância entre a face do incisivo central superior e a face do incisivo central inferior com os dentes em oclusão.
- Trespasse vertical: distância entre a borda do incisivo central inferior e a borda do incisivo central superior com os dentes em oclusão.
- Abertura oral máxima: distância entre as faces incisais dos dentes incisivos centrais (superior e inferior), acrescida da medida do trespasse vertical;
- Lateralização mandibular: distância horizontal entre as linhas dos incisivos superiores e inferiores após deslizamento lateral da mandíbula (para a direita e depois para a esquerda), realizando o ajuste pertinente em casos de desvio de linha média dos incisivos centrais;
- Protrusão mandibular: distância horizontal entre a face vestibular dos dentes incisivos centrais (superior e inferior) após deslizamento anterior, acrescido pelo trespasse horizontal.

### Avaliação da musculatura supra hioidea - Eletromiografia de Superfície (EMGs)

Os exames de EMGs foram realizados pelo mesmo fonoaudiólogo, nas mesmas condições ambientais. A avaliação eletromiográfica dos músculos suprahióideos dos participantes foi realizada com base em metodologia específica^(23)^. Para tanto, foi utilizado equipamento (Miotool 400) com 4 canais, sendo todos os canais calibrados da seguinte forma: em 500 microvolts (μV) com filtro do tipo passa-banda (20-500 Hz) e do tipo notch (60 Hz) e ganho de 100 vezes, com baixo nível de ruído (< 5μV RMS). O software utilizado para a captação e processamento do exame de EMGs foi o aplicativo Miograph 2.0 do fabricante Miotec® Equipamentos Biomédicos que faz aquisição, armazenamento e processamento on-line de sinais e é executado sob sistema operacional Windows XP ou superior. Os sinais da atividade elétrica dos movimentos musculares foram captados por eletrodos bipolares de superfície Ag/AgCl, descartáveis, modelo SDS500, duplos, fixados com fita transpore (3M).

O posicionamento dos eletrodos ocorreu diretamente na pele, previamente limpa com álcool 70% e após tricotomia, obedecendo à técnica de colocação do ponto médio do ventre muscular na direção longitudinal do feixe muscular na posição mesodistal do músculo, onde observa-se maior amplitude de sinal para este tipo de eletrodo. sugerido por Soderberg e Cook^(24)^, após a colocação dos eletrodos, foi realizado teste e, se necessário reposicionados. Os sinais captados foram analisados em root mean square (RMS) e expressos em microvolts (μV). O cabo de referência (cabo terra) foi conectado ao eletrodo e fixado sobre o pulso direito.

Para a coleta, os participantes permaneceram sentados confortavelmente em uma cadeira, com as costas apoiadas, pés apoiados no chão, mãos apoiadas nos membros inferiores, cabeça posicionada adequadamente (Plano de Frankfurt paralelo ao chão), olhos abertos e buscando um ponto fixo pré-determinado. Todos os indivíduos foram orientados quanto ao teste.

A avaliação eletromiográfica foi realizada em três etapas e com intervalos de 3 minutos entre os volumes:

- Repouso - cada participante foi instruído a permanecer o mais relaxado possível durante 1 minuto. Após esse tempo foram gravadas três coletas de 30 segundos de atividade muscular dos músculos supra hioideos;
- Avaliação da deglutição de água em temperatura ambiente: todos os participantes receberam a seguinte instrução: “Beba toda a água em apenas um gole”. Todos os dados foram gravados em janelas de 15 segundos. Foram realizados os seguintes testes por três vezes:
  o Deglutição voluntária de saliva. Os participantes receberam a seguinte instrução - “Engula a saliva que está agora na sua boca”;
  o Oferta de água na seringa com 10 ml de água;
  o Oferta de água na seringa com 16,5 ml de água;
  o Oferta de água na seringa com 20 ml de água.
- Avaliação da deglutição em maior volume (50ml) e em auto oferta em copo: Todos os participantes receberam a seguinte instrução: “Beba toda a água como faz naturalmente, mas mantendo a mesma postura com a cabeça”. Todos os dados foram gravados em janelas de 15 segundos.

Para a análise dos resultados eletromiográficos, foi considerada a característica da amplitude do sinal. Na situação de repouso, os valores obtidos representam a média (RMS) da atividade eletromiográfica observada em 30 segundos. A duração da atividade muscular durante as tarefas de deglutição foi obtida pela seleção do trecho representativo da ativação muscular (situação *on, pico* e *off*).

Em decorrência da conhecida variabilidade do sinal eletromiográfico^(25)^ faz-se necessária a utilização de técnicas de normalização dos dados para a comparação entre grupos, sendo esta uma forma de transformação dos valores absolutos da amplitude em valores relativos referentes a um valor de amplitude caracterizado como 100%. A técnica adotada no presente estudo foi a normalização das atividades em relação à tarefa de repouso.

Foi realizada a análise de confiabilidade a fim de determinar o índice de concordância entre os examinadores e assim, garantir maior fidedignidade das medidas. Para tanto, foram selecionadas randomicamente 50 amostras eletromiográficas referentes às tarefas de deglutição de um total de 615 disponíveis (3 sujeitos apresentaram sinal eletromiográfico com artefatos, impossibilitando a coleta). Essas amostras foram analisadas, independentemente, por dois fonoaudiólogos com experiência na área, cegos ao estudo. O coeficiente de correlação mostrou-se alto para todas as comparações (intervalo de confiança de 95% [IC] = 0.8345-0.9158), indicando alta consistência entre os examinadores.

### Mensuração da Pressão de Língua (PLL Pró-Fono):

Foi utilizado o equipamento nacional de *Biofeedback* Pró-Fono - Pressão de Lábios e de Língua (PLL Pró-Fono), liberado pela Agência Nacional de Vigilância Sanitária (ANVISA) para uso clínico no Brasil. O PLL Pró-Fono é formado por um sensor de pressão conectado a uma placa eletrônica e acondicionado em um gabinete plástico. Neste gabinete, é conectado um tubo plástico flexível de ar que recebe um bulbo descartável com conector macho em sua extremidade para a coleta da pressão de língua, conforme as instruções contidas no manual técnico. As informações de variação na pressão exercida sobre o bulbo são enviadas pelo dispositivo para o *software* do equipamento instalado no computador, que fornece *feedback* visual por meio de gráficos em tempo real. No computador, é necessária a instalação prévia do *software* PLL Pró-Fono, que tem como requisito o sistema operacional *Windows XP* ou superior.

A avaliação da pressão de ponta e dorso de língua foi realizada conforme orientações abaixo:

- Pressão de dorso de língua - foi solicitado que o paciente segurasse a haste do bulbo de ar com uma das mãos, abrisse a boca e colocasse o bulbo de ar sobre o dorso da língua, de forma que a extremidade do bulbo de ar ligada à sua haste coincidisse com a ponta da língua do usuário e que a pressão entre o dorso da língua e o palato fosse realizada nas hemifaces do bulbo de ar. O paciente poderia ocluir levemente os lábios, mas sem contato ou pressão entre os dentes, e foi orientado e incentivado a pressionar toda a extensão da língua no bulbo de ar contra a região palatina durante 5 segundos, com descanso de 30 segundos. Foram realizadas 3 coletas e em cada intervalo, o paciente poderia deglutir a saliva e secar a cavidade oral com gaze, para evitar que o bulbo deslizasse durante o exame.
- Pressão de ponta de língua - foi solicitado que o paciente segurasse a haste do bulbo de ar com uma das mãos, abrisse a boca e colocasse o bulbo de ar sobre a ponta da língua, de forma que a extremidade do bulbo de ar ligada à sua haste coincidisse com os alvéolos dos dentes incisivos centrais do usuário e que a pressão entre a ponta da língua e o palato fosse realizada nas hemifaces do bulbo de ar. O paciente poderia ocluir levemente os lábios, mas sem contato ou pressão entre os dentes, e foi orientado e incentivado a pressionar toda a ponta da língua no bulbo de ar contra a região de papila palatina durante 5 segundos, com descanso de 30 segundos. Foram realizadas 3 coletas e em cada intervalo, o paciente poderia deglutir a saliva e também secar a cavidade oral com gaze, para evitar que o bulbo deslizasse durante o exame.

## ANÁLISE DE DADOS

Os dados coletados foram submetidos à análise estatística no *software* SPSS versão 28.0, sendo realizadas duas formas de análise. Inicialmente, os dados receberam análise descritiva, para a caracterização da amostra: as variáveis quantitativas foram descritas pela média, desvio padrão, mediana, mínimo e máximo; e as variáveis qualitativas foram descritas pela contagem total e porcentagem. A análise inferencial para investigar a presença de correlação entre as medidas de pressão de língua (dorso e ponta) e as demais variáveis estudadas foi realizada pelo coeficiente de correlação de *Spearman.* Trata-se de um teste não-paramétrico, que é adequado e uma alternativa viável para esta análise, pois não exige normalidade ou igualdade das variâncias e é aplicável a amostras pequenas. A interpretação dos dados da análise de correlação seguiu os parâmetros: relação negativa forte (r < -0,750); relação negativa moderada (-0,750 < r < -0,500); relação negativa fraca (-0,500 < r < -0,250); baixa ou nenhuma relação (-0,250 < r < 0,250); relação positiva fraca (0,250 < r < 0,500); relação positiva moderada (0,500 < r < 0,750); relação positiva forte (r > 0,750).

## RESULTADOS

A caracterização da amostra de pacientes com relação às variáveis demográficas e clínicas encontra-se descrita na Tabela 1. Foi possível observar que a média de idade dos participantes foi de 34,7 anos, o grupo com maior número de participantes foi aquele com diagnóstico de paralisia facial seguido pelo diagnóstico de fissura labiopalatina e a maioria dos participantes apresentou a classificação da mordida como Classe I de Angle.

**Tabela 1:** Caracterização da amostra segundo dados demográficos e clínicos (n=44)

	Resultados
**Idade, em anos**	
n válido (dados disponíveis)	44
média (±DP)	34,7 (±13,4)
mediana (min; max)	35,0 (17; 63)
**Sexo, n (%)**	
Masculino	20 (45,5%)
Feminino	24 (54,5%)
**Patologia, n (%)**	
Paralisia Facial	13 (29,5%)
Fissura Labiopalatina	11 (25,0%)
Trauma de Face	6 (13,6%)
SAHOS	3 (6,8%)
Disfagia	9 (20,5%)
Cirurgia ortognática	2 (4,5%)
**Oclusão de Angle, n (%)**	
Classe I	35 (79,5%)
Classe II	2 (4,5%)
Classe III	7 (15,7%)

Legenda: n: número de participantes; DP: desvio padrão; min.: mínimo; max.: máximo; SAHOS: Síndrome da Apneia e Hipopneia Obstrutiva do Sono

Os dados referentes às medidas antropométricas faciais e às medidas dinâmicas da amplitude mandibular, assim como a pontuação no protocolo AMIOFE-E encontram-se descritas na Tabela 2. Para fins de comparação foi incluído na tabela as medidas de normalidade descritas na literatura^(3,20,21)^. No presente estudo, foi possível observar uma discrepância entre os terços da face dos participantes, sendo o terço inferior mais alongado. Quanto às medidas de amplitude mandibular, observou-se maior comprometimento das medidas de lateralização de mandíbula e protrusão de mandíbula, e da medida de trespasse vertical.

**Tabela 2:** Caracterização da amostra segundo às medidas faciais, dinâmicas e do protocolo AMIOFE-E (n=44)

		**Medida, em mm**		**Normalidade, em mm**
		**média (±DP)**	**mediana (min; max)**	**média (±DP)**
**Medidas faciais e mandibulares**	**Terço superior da face**	58,5 (±7,6)	58,0 (41,6; 72,6)	55,00 - 65,00
	**Terço médio da face**	58,2(±4,6)	58,3 (48,6; 66,9)	55,00 - 65,00
	**Terço inferior da face**	65,6 (±6,7)	66,1 (53,1; 86,4)	55,00 - 65,00
	**Abertura máxima**	45,0 (±9,9)	46,7 (9,2; 58,5)	40,00 - 60,00
	**Lateralização da mandíbula - lado direito**	6,0 (±3,1)	6,2 (0,0; 13,9)	7,00 - 11,00
	**Lateralização da mandíbula - lado esquerdo**	6,1 (±3,0)	6,2 (0,1; 13,6)	7,00 - 11,00
	**Protrusão da mandíbula**	6,5 (±2,6)	6,5 (0,0; 11,1)	7,00 - 11,00
	**Trespasse horizontal**	2,8 (±4,0)	3,5 (-13,7; 9,1)	2,00 - 3,00
	**Trespasse vertical**	2,7 (±2,5)	2,8 (-7,8; 7,3)	1,00 - 2,00
		**Pontuação do paciente**		**Pontuação Máxima Possível**
		**média (±DP)**	**mediana (min; max)**	
**AMIOFE-E**	**Aparência e condição postural / posição - total**	53,1 (±4,1)	53,0 (45; 61)	64
	**Mobilidade - total**	91,2 (±17,7)	91,0 (52; 160)	114
	**Funções - total**	42,3 (±5,6)	42,0 (29; 55)	52
	**AMIOFE - pontuação final**	184,4 (±20,9)	184,0 (134; 258)	230

**Legenda:** n: número de participantes; mm: milímetros; DP: desvio padrão; min.: mínimo; max.: máximo; AMIOFE-E: Protocolo de Avaliação Miofuncional Orofacial com Escores Expandido

Para o AMIOFE-E foi incluído na Tabela 2 a pontuação máxima possível, segundo o próprio teste. Segundo esta análise, considerando a média de pontuação obtida para o grupo de participantes, observa-se que: para a aparência e condição postural a amostra atingiu 82,9% da pontuação esperada, para mobilidade 80%, para funções de 81,3% e para o total 80,2%.

Com relação aos resultados da avaliação eletromiográfica da musculatura suprahioidea, tanto no repouso quanto na deglutição de saliva e de volumes controlados de água (10; 16,5; 20; e 50ml), encontra-se descrita na Tabela 3. Cabe ressaltar que o número de picos encontrados na deglutição da saliva e dos volumes de água corresponde ao número de deglutições realizadas pelos participantes para deglutir cada volume. Conforme esperado, a ativação/recrutamento muscular durante a deglutição foi mais evidente para deglutição de 50ml de água, havendo necessidade de mais tempo para concluir a tarefa e necessitando de um número maior de deglutições para finalizar completamente o volume ofertado.

**Tabela 3:** Caracterização da amostra segundo os resultados da avaliação eletromiográfica (n=41)

**Variável**			**Resultado da EMG**		
			n válido	Média (DP)	Mediana (min;max)
Repouso: atividade eletromiográfica, em μV (RMS)		Lado direito	41	5,2 (±6,2)	3,6 (0,0; 33,9)
		Lado esquerdo		3,8 (±2,9)	3,4 (0,0; 14,5)
Tempo, em segundos		Deglutição de saliva	41	1,0 (±0,5)	1,1 (0,0; 2,2)
		Deglutição de 10ml de água		1,1 (±0,6)	1,0 (0,0; 3,3)
		Deglutição de 16,5ml de água		1,0 (±0,5)	1,0 (0,0; 2,3)
		Deglutição de 20ml de água		1,1 (±0,5)	1,1 (0,0; 2,9)
		Deglutição de 50ml de água		8,0 (±4,2)	7,8 (0,0; 16,7)
Número de picos		Deglutição de saliva	41	1,0 (±0,5)	1,0 (0,0; 2,0)
		Deglutição de 10ml de água		1,1 (±0,5)	1,0 (0,0; 2,0)
		Deglutição de 16,5ml de água		1,0 (±0,4)	1,0 (0,0; 1,7)
		Deglutição de 20ml de água		1,1 (±0,4)	1,0 (0,0; 2,0)
		Deglutição de 50ml de água		4,5 (±2,3)	4,3 (0,0; 10,7)
Medida normalizada: atividade eletromiográfica, em μV (RMS)	Lado direito	Deglutição de saliva	41	7,5 (±4,0)	6,9 (1,2; 18,3)
		Deglutição de 10ml de água		8,2 (±5,0)	7,1 (0,0; 20,8)
		Deglutição de 16,5ml de água		8,5 (±5,1)	8,1 (0,0; 21,2)
		Deglutição de 20ml de água		9,2 (±4,9)	10,0 (1,2; 22,7)
		Deglutição de 50ml de água		6,3 (±3,6)	6,2 (0,0; 15,3)
	Lado esquerdo	Deglutição de saliva		81 (±4,0)	8,2 (1,8; 18,3)
		Deglutição de 10ml de água		8,9 (±4,7)	8,6 (0,0; 21,3)
		Deglutição de 16,5ml de água		9,1 (±4,7)	8,8 (0,0; 20,1)
		Deglutição de 20ml de água		9,6 (±4,3)	9,8 (1,7; 22,6)
		Deglutição de 50ml de água		6,7 (±3,2)	6,9 (0,0; 14,0)

**Legenda:** n: número de participantes; DP: desvio padrão; min.: mínimo; max.: máximo; EMG: eletromiografia de superfície; RMS: *root mean square*; μV: microvolts

A caracterização da amostra de participantes com relação aos resultados da avaliação da pressão de língua encontra-se descrita na Tabela 4. Observou-se que a pressão de língua foi semelhante entre o dorso e a ponta.

**Tabela 4:** Caracterização da amostra segundo a avaliação da pressão de língua (n=44)

	**Medição, em kPa**		
	n válido (dados disponíveis)	média (±DP)	mediana (min; max)
**Dorso de língua**	43	33,6 (±14,3)	32,8 (0,0; 68,6)
**Ponta de língua**		33,6 (±12,0)	33,5 (10,6; 62,7)

**Legenda:** n: número de participantes; DP: desvio padrão; min.: mínimo; max.: máximo; kPa: Kilopascal.

A Tabela 5 apresenta o resultado da análise de correlação entre os dados da avaliação da pressão de língua e as demais variáveis estudadas. Foram encontradas as seguintes correlações significativas: entre a pressão de dorso de língua e a medida do terço inferior da face; entre a pressão do dorso de língua e as medidas de trespasse vertical e horizontal entre a pressão de dorso de língua e a pontuação de aparência e condição postural no protocolo AMIOFE-E^(3)^ entre a pressão de dorso de língua e a pontuação para as funções no protocolo AMIOFE-E^(3)^ e entre as pressões de dorso e ponta de língua. Cabe ressaltar, em todas as correlações encontradas foram fracas e um dos sujeitos da amostra não conseguiu realizar as coletas devido à hipersensibilidade com reflexo nauseoso persistente durante as tentativas do exame.

**Tabela 5:** Correlações entre os resultados da avaliação de pressão de língua e as demais variáveis de caracterização da amostra

		**Correlações com a avaliação da pressão de língua**			
		**Dorso de língua**		**Ponta de língua**	
		**r**	**p-value**	**r**	**p-value**
Idade		-0,125	0,426	-0,241	0,120
Sexo		0,103	0,513	-0,054	0,731
Patologia		-0,146	0,352	0,068	0,665
Medida	Terço superior da face	0,071	0,650	0,060	0,702
	Terço médio da face	0,069	0,662	0,094	0,550
	Terço inferior da face	***-0,306***	0,046*	-0,018	0,911
	Oclusão de Angle	-0,159	0,310	0,265	0,086
	Abertura máxima	0,121	0,470	-0,092	0,581
	Desvio de linha média	-0,053	0,747	0,067	0,687
	Lateralização - direita	-0,083	0,620	-0,301	0,067
	Lateralização - esquerda	0,110	0,511	0,076	0,650
	Protrusão da mandíbula	0,240	0,152	-0,214	0,203
	Retração da mandíbula	-0,198	0,241	0,033	0,847
	Trespasse horizontal	***0,329***	0,047*	-0,108	0,525
	Trespasse vertical	***0,418***	0,010*	-0,038	0,824
Pontuação AMIOFE	Aparência e condição postural	***0,471***	***0,001*** *	0,036	0,817
	Mobilidade	0,014	0,930	0,031	0,842
	Funções	***0,365***	0,016*	0,113	0,471
	Pontuação total	-0,001	0,994	-0,145	0,355
Avaliação EMG	Atividade em repouso - direita	0,139	0,375	-0,057	0,715
	Atividade em repouso - esquerda	0,176	0,259	-0,016	0,919
	Tempo de deglutição - saliva	0,132	0,398	-0,013	0,935
	Tempo de deglutição - 10ml	-0,157	0,315	-0,034	0,830
	Tempo de deglutição - 16,5ml	-0,156	0,317	-0,024	0,878
	Tempo de deglutição - 20ml	0,086	0,582	0,035	0,822
	Tempo de deglutição - 50ml	-0,279	0,070	-0,203	0,193
	Número de picos - saliva	0,206	0,184	0,147	0,347
	Número de picos - 10ml	-0,212	0,172	0,022	0,890
	Número de picos - 16,5ml	-0,125	0,425	0,052	0,738
	Número de picos - 20ml	-0,007	0,967	-0,053	0,738
	Número de picos - 50ml	-0,265	0,085	-0,056	0,722
	Pico máximo - saliva - direita	***0,355***	0,019*	0,192	0,217
	Pico máximo - 10ml - direita	-0,103	0,509	0,063	0,689
	Pico máximo - 16,5ml - direita	-0,091	0,563	0,069	0,659
	Pico máximo - 20ml - direita	-0,017	0,916	0,065	0,677
	Pico máximo - 50ml - direita	0,021	0,892	-0,047	0,767
	Pico máximo - saliva - esquerda	***0,329***	0,031*	0,167	0,285
	Pico máximo - 10ml - esquerda	-0,118	0,451	0,119	0,445
	Pico máximo - 16,5ml - esquerda	-0,118	0,452	0,105	0,503
	Pico máximo - 20ml - esquerda	-0,050	0,748	0,110	0,482
	Pico máximo - 50ml - esquerda	-0,082	0,599	0,078	0,618
	Normalizada - saliva - direita	0,185	0,247	0,169	0,292
	Normalizada - 10ml - direita	0,059	0,715	0,116	0,469
	Normalizada - 16,5ml - direita	0,032	0,844	0,103	0,522
	Normalizada - 20ml - direita	0,125	0,435	0,088	0,585
	Normalizada - 50ml - direita	0,129	0,421	0,107	0,504
	Normalizada - saliva - esquerda	0,034	0,835	-0,010	0,953
	Normalizada - 10ml - esquerda	-0,045	0,780	-0,037	0,817
	Normalizada - 16,5ml - esquerda	-0,069	0,668	-0,057	0,723
	Normalizada - 20ml - esquerda	0,007	0,965	-0,085	0,598
	Normalizada - 50ml - esquerda	0,000	1,000	-0,018	0,910
Medição da pressão de dorso de língua (PLL)		-	-	***0,490***	<0,001*

* diferença estatisticamente significativa, de acordo com o coeficiente de correlação de Spearman

**Legenda:** r: coeficiente de correlação; EMG: eletromiografia de superfície; PLL: pressão de lábios e língua

## DISCUSSÃO

De maneira geral, a análise dos resultados do presente estudo apontou as seguintes correlações significativas entre os instrumentos utilizados para a caracterização da amostra de participantes: a pressão de língua foi maior quanto maior o trespasse horizontal e melhor pontuação na avaliação miofuncional nos aspectos de repouso e nas funções orofaciais; e quanto maior a medida do terço inferior, menor a pressão de dorso de língua.

Apesar de quase a totalidade da amostra de paciente incluídos no presente estudo ter a oclusão classificada como Classe I, existem pacientes que tiveram a oclusão classificada como Classe II e III^(26)^, fato que interferiu nos resultados da avaliação clínica de motricidade orofacial e nas medidas antropométricas e de amplitude mandibular. Muitos estudos^(27-29)^ já foram realizados relacionando a forma com a função do sistema estomatognático.

Considerando as maloclusões de Classe III, a literatura aponta que é frequente encontrar nesta população alteração mastigatória, com predominância de movimentos verticalizados e uso do dorso da língua para amassamento do alimento; lábios flácidos e entreabertos no repouso; língua flácida, alargada e em assoalho bucal^(27,30)^. Conforme observado no presente estudo, essas foram as duas áreas mais comprometidas na avaliação da motricidade orofacial dos participantes incluídos na pesquisa. A maior incidência de maloclusão de Classe III no grupo com fissuras labiopalatinas está relacionada tanto ao crescimento e avanço mandibular ao longo do desenvolvimento quanto à hipoplasia maxilar, que ocorre devido à alteração óssea congênita e às múltiplas abordagens cirúrgicas na região^(31)^.

Nas maloclusões de Classe II e III, foi possível observar alterações nas medidas dos trespasses tanto horizontal quanto vertical, sendo que na maloclusão de Classe III, o trespasse horizontal foi bastante reduzido e nos casos mais graves, houve inversão da relação maxilomandibular típica. Conforme observado no presente trabalho, esses pacientes tendem a apresentar menor força de língua quanto maior for a redução do trespasse horizontal. Este achado corrobora um estudo anterior^(3)^, que também analisou a força de língua. Segundo os autores deste estudo, indivíduos com maloclusão de Classe III apresentam menor força de língua e maior tempo de pressão durante a deglutição de alimento pastoso homogêneo se comparados a sujeitos controle com oclusão de classe I e sem queixas ou intervenções orofaciais prévias^(32)^. Cabe ressaltar que os dados relacionados às alterações de pressão de língua e às maloclusões não são uniformes na literatura. O estudo realizado por Silva et al.^(33)^ não encontrou relação significante entre a força de língua e os diferentes tipos de oclusão dentária. Com base nos resultados encontrados no presente estudo, uma possível explicação para a menor pressão de língua observada nos indivíduos com maloclusão de classe III seria possivelmente que a projeção da mandíbula, característica desta população, tem como consequência um aumento no espaço intraoral, exigindo maior amplitude de movimentos da língua para a realização de contra resistência em região de palato (pressão)^(34)^. Apesar de estudo prévio apontar que o tipo de oclusão (I, II e III de Angle) não interfere na força da língua^(33)^, sabe-se que nas más oclusões de Classe III, tanto a língua quanto o osso hioide se encontram anteriorizados^(28)^. Ao exercer os movimentos para a deglutição, a língua de indivíduos com maloclusão de Classe III segue o mesmo padrão de movimentos que observados na Classe I, mas com menor força e necessidade de maior tempo para realizar a mesma atividade, tornando a função menos efetiva^(28)^.

Outro dado que merece destaque foi a desproporção facial observada nos participantes incluídos no presente estudo, sendo que, de maneira geral, o terço inferior da face mostrou-se mais alongado. Novamente, esse resultado pode ser explicado pela interferência dos dados obtidos para o grupo de pacientes com maloclusão de Classe III. Segundo a literatura, essa população tende a apresentar como característica uma face mais longa (dólicofacial), com flacidez da musculatura orofacial, levando a alterações das funções orofaciais, principalmente de mastigação e respiração^(26)^.

A avaliação clínica do sistema miofuncional orofacial realizada através do protocolo AMIOFE-E^(32)^ apresentou escores próximos a 80% em todos os campos de análise, sendo que dois desses campos apresentaram correlação positiva com a força do dorso da língua: a condição postural e as funções orofaciais (respiração, mastigação e deglutição são contempladas no protocolo). A força de língua à avaliação clínica miofuncional corrobora a hipótese de que as estruturas orofaciais e as suas funções se interrelacionam^(27,35)^. O mesmo é válido para as diferentes regiões da língua. No presente estudo, os resultados indicaram que não houve diferença entre a pressão de ponta e de dorso de língua. Estudo anterior^(24)^, que utilizou um dinamômetro para medir a pressão de ponto e de dorso de língua, encontrou outro resultado: a ponta da língua apresentou menor força do que o dorso. Segundo os autores, a diferença encontrada pode ser explicada pelos diferentes tipos de fibra muscular em cada uma das regiões da língua - fibras do tipo II na ponta, garantindo uma rápida contração e fibras do tipo I no dorso, oferecendo uma contração mais lenta e resistente. Em contrapartida, outro estudo^(28)^, que utilizou sensores em palato para verificar a pressão de língua, descreve que a ponta da língua é a primeira porção do órgão a pressionar o palato duro e uma das últimas a relaxar após a deglutição, mantendo a pressão fixa na papila palatina. É importante ressaltar que a variabilidade metodológica dos estudos e de uso de equipamentos para medir a pressão de língua dificulta a comparação e generalização dos resultados.

A função de deglutição, avaliada por meio da EMGs de músculos supra hioideos, não apresentou variação de tempo ou número de picos nas deglutições controladas (saliva, 10ml, 16,5ml e 20ml). Apenas na deglutição em goles livres foi observado valores diferentes, devido à possibilidade de fracionar o conteúdo, ingerindo-o em volumes menores. Foi observada uma curva crescente bilateralmente nos valores de pico máximo eletromiográfico e na medida normalizada, indicando que o recrutamento de fibras musculares aumenta conforme o volume a ser ingerido aumenta também. Este achado é o inverso dos encontrados em estudos recentes, realizados com articulografia magnética^(35,36)^. Em um destes estudos^(35)^, sensores em palato foram associados para a mensuração da pressão de língua durante a deglutição de diferentes volumes de água (3 e 10 ml), no qual foi verificada uma mudança na movimentação do dorso da língua com volumes menores do bolo antes da deglutição. Os autores indicam como justificativa para essa maior ativação dos músculos da língua, os movimentos de rotação do dorso da língua para posicionar o bolo pequeno sobre o centro da língua para a deglutição. Os autores mencionaram ainda que a tendência é um menor recrutamento dos músculos da língua para a tarefa com o aumento do volume, pois volumes maiores se acomodam mais facilmente no centro da língua. Nesse estudo, não houve aumento significativo da pressão exercida pela língua no palato^(36)^.

Apenas a deglutição de saliva (menor volume) apresentou correlação estatisticamente significante com a força de língua. Em um estudo que avaliou a morfologia dentofacial e a função da língua durante a deglutição em indivíduos jovens^(27)^ os autores concluíram que a deglutição de saliva é a função que apresenta maior efeito na morfologia dentofacial, o que pode explicar o achado sobre deglutição de saliva nesta pesquisa.

Deve-se considerar que todos os métodos de avaliação padronizada apresentam uma vantagem considerável no processo terapêutico: a possibilidade de comparação intra e intersujeito. Além disso, é importante considerar que a avaliação clínica da motricidade orofacial é o método mais prático e acessível a todos os fonoaudiólogos clínicos e os seus achados devem ser correlacionados com as demais formas de avaliação.

Por fim, o presente estudo apresentou algumas limitações. Como já apontado, o pequeno número de participantes, a heterogeneidade das patologias e a ausência de grupo controle limitou a generalização dos resultados. Outra limitação se refere ao equipamento utilizado para verificação da pressão de língua. Durante a coleta de dados, foi possível constatar a dificuldade dos pacientes em manter o bulbo de ar (utilizado para realização da mensuração da pressão) estável dentro da cavidade oral. Além disso, o bulbo mostrou-se grande em relação ao espaço intraoral, provocando náusea em diversos dos participantes e, portanto, interferindo na coleta dos dados. O PLL fornece a média de pressão da língua em 5 segundos e não o pico máximo da pressão conforme outros equipamentos existentes no mercado para o mesmo fim. Esse fato impossibilitou a comparação dos resultados do presente estudo aos resultados encontrados na literatura em pesquisas semelhantes. Estudo futuro deve ser realizado com um maior número de participantes divididos pelo tipo de maloclusão, a fim de confirmar os resultados encontrados.

## CONCLUSÃO

O resultado do presente estudo encontrou as seguintes correlações significativas entre os instrumentos utilizados para a caracterização da amostra de participantes: a pressão de língua foi maior quanto maior o trespasse horizontal e melhor pontuação na avaliação miofuncional nos aspectos de repouso e nas funções orofaciais; e quanto maior a medida do terço inferior, menor a pressão de dorso de língua. Os resultados ainda sugerem que as alterações miofuncionais orofaciais encontradas nos diferentes grupos de pacientes estão mais relacionadas às discrepâncias maxilomandibulares do que às patologias pesquisadas propriamente ditas.
